# Effective factors on Sharp Score in patients with rheumatoid arthritis: a retrospective study

**DOI:** 10.1186/s12891-021-04742-3

**Published:** 2021-10-09

**Authors:** Jianting Wen, Jian Liu, Ling Xin, Lei Wan, Hui Jiang, Yue Sun, Yanqiu Sun, Xin Wang, Jie Wang

**Affiliations:** 1 Anhui University of Traditional Chinese Medicine, Hefei, 230031 Anhui Province China; 2 Department of Rheumatology and Immunology, First Affiliated Hospital of Anhui University of Traditional Chinese Medicine, Hefei, 230038 Anhui Province China; 3 Institute of Rheumatology, Anhui College of Traditional Chinese Medicine, Hefei, 230038 Anhui Province China; 4 Key Laboratory of Xin’an Medicine of the Ministry of Education, Anhui University of Chinese Medicine, Hefei, Anhui 230038 P. R. China; 5 Anhui Province Key Laboratory of Modern Chinese Medicine Department of Internal Medicine Application Foundation Research and Development, Hefei, 230031 Anhui China

**Keywords:** Sharp score, Rheumatoid arthritis, Effective factors, Retrospective study

## Abstract

**Background:**

This study aims to describe the association between sharp score and clinical indexes, bone metabolism indexes, Disease Activity Score (DAS28) and sociodemographic factors in rheumatoid arthritis (RA).

**Methods:**

Data were collected from the HIS (hospital information system), a national inpatient database in China, with information on the patients hospitalized during the period from 2012 to 2019. The association between sharp score and effective factors were identified using multinomial logistic regression and association rule mining (ARM).

**Results:**

Three thousand eight hundred and forty patients were included: 82.66% males, 17.34% females, mean (SD) age 56.95 (12.68) years and symptom duration 3.45 (1.09) years. Spearman correlation analysis and Association rules analysis showed that there were significant positive correlations between sharp score and effective factors. Logistic regression analysis presented that erythrocyte sedimentation rate (ESR), high-sensitivity C-reactive protein (CRP), rheumatoid factor (RF) were risk factors of sharp score. In the analysis of individual outcomes, sex, age, symptom duration, DSA28 score, RF, ever drinker, and radiographic grading of hands were influence factors of sharp score.

**Conclusion:**

Sharp score should be taken into consideration in formulating treatment strategies in RA.

## Background

Rheumatoid arthritis (RA) is a chronic inflammatory disease characterized by synovial membrane inflammation [1, 2]. Erosive joint damage and bone destruction are the most common manifestation of RA, which might induce ankylosis, malformation, even loss of normal joint function [3, 4]. Current goals of treatment in RA include achieving disease remission, reducing functional disability as well as minimizing pain [5, 6]. Erosions are the hallmark of bone destruction in RA [7, 8]. Controlling joint destruction and bone destruction have become the major objective for treating RA, because radiographic joint damage correlates strongly with long-term functional decline in RA patients [9, 10]. Radiographic grading of hands is the most commonly used method for the evaluation of different levels of bone erosion in clinical practice [11, 12]. Bone erosion score (vdH sharp score) has also been used to measure morphological parameters that quantify the bone erosion and bone destruction, giving useful information for early detection and early treatment of RA [13, 14]. Few studies to date have studied the overall impact of RA on sharp score and its effective factors. In addition, there has been no study that looks for effective factors associated with sharp score of RA on the basis of large data by doing the mining and analysis of this data.

This study retrospectively analyzed the clinical data of enrolled patients to investigate the value of sharp score and its effective factors in RA. The Spearman correlation analysis, Association rules analysis and Logistic regression analysis are methods of analysis that allows for the identification of risk factors associated with sharp score. Using these three methods, this study aims to: (a) sharp score exhibits diagnostic value for RA; and (b) analyze the role of effective factors as determinants of sharp score in RA.

## Methods

### Study design

We conducted this retrospective cohort study in a population of RA patients. RA was defined based on the classification criteria revised by the ACR/EULAR (American College of Rheumatology/European League Against Rheumatism) criteria in 2010 [15]. This study was reviewed and approved by the institutional committee of the First Hospital Affiliated to the Anhui University of Chinese Medicine on research ethics, and conforms to the ethical guidelines of the 1975 Declaration of Helsinki. All patients provided written informed consent for inclusion in this study.

### Study population and data collection

A total of 3840 RA patients visited the First Hospital Affiliated to the Anhui University of Chinese Medicine from January 2012 to December 2019. During the follow-up, accumulated demographic and laboratory data obtained from patients’ electronic medical records were longitudinally examined. The following clinical data were obtained from all participants: age; sex; laboratory data, including ESR, CRP, RF, anti-cyclic citrullinated peptide antibody (CCP), immunoglobulins A (IGA), immunoglobulin G (IGG), immunoglobulin M (IGM), complement 3 (C3), and complement 4 (C4), bone alkaline phosphatase (BALP), osteocalcin (OC), Osteoprotegerin (OPG), receptor activator for nuclear factor-κB ligand (RANKL); DAS28 score, and sharp score.

### Radiographic evaluation

Radiographs of the hands were assessed according to the Sharp method. In total, scores for the 3840 radiographs (from 3840 RA patients) were determined by two experienced rheumatologist who was blinded to the clinical data. Sixteen areas were considered for assessing erosions and joint space narrowing (JSN) for the hands. The maximum erosion score of the hands and wrists was 160. Accordingly, the maximum JSN score of the hands and wrists was 120. The sum of the erosion and JSN scores is the total Sharp/van der Heijde score (SHS) (maximum: 280). Therefore, radiographic joint destruction was quantified as the total SHS score divided by the duration of RA.

### Statistical analysis

Descriptive statistics were used to summarize the demographic characteristics of the cohort. Continuous variables were expressed as mean ± standard deviation or median (interquartile range). The correlations between sharp score and clinical indexes during the study period were estimated using Spearman’s correlation coefficients. The associations of important covariates with sharp score were examined using the binary linear regression model and association rule mining (ARM) [16]. Analyses were performed using SPSS version 15.0 (SPSS Chicago, Ill, USA) and GraphPad software (version 8.0).

## Results

### Characteristics of the study population

The study sample was composed of 3840 RA patients, with a mean age of 56.95 years (standard deviation 12.68, range 18–95) and of whom 82.66% were female. The median number of sharp score was 20.00 (IQR: 7.00, 56.00). 3003 (78.15%) were seropositive for either RF and 3636 (94.68%) were seropositive for CCP. The mean (SD) symptom duration was 3.45 (1.09) years and the mean (SD) disease duration was 6.92 (1.20) years. The main characteristics of the study population are detailed in Table 1.

**Table 1:** Characteristics of study population (*n* = 3840)

**Quantitative Variables**	**Mean**	**Standard Deviation**
Age (years)	56.95	12.68
Symptom duration (years)	3.45	1.09
Disease duration (years)	6.92	1.20
BMI (Kg/m^2^)	22.80	4.51
Tender joint count, 0–28	10.73	5.63
	**Subjects**	**Percentage**
Sharp score		
≤ 0 score	111	2.89
> 0 score, ≤ 50 score	2674	69.64
> 50 score	1055	27.47
Gender		
Male	666	17.34
Female	3174	82.66
Ever smoker	458	11.93
Ever drinker	582	15.16
RF positivity	3001	78.15
CCP positivity	3636	94.68
Presence of radiographic erosions	2162	56.32
Prednisolone use	2150	55.98
DMARD treatment (at baseline)		
DMARD-naive	3017	78.56
MTX monotherapy	1368	35.62
Non-MTX csDMARD	888	23.13
Combination csDMARD	905	23.56
Education status		
None or primary	1545	40.23
Secondary or vocational	1740	45.31
Tertiary	555	14.45
Housing status		
Private housing	2397	62.42
Government housing	1443	37.58
Employment status		
Currently employed	1735	45.18
Unemployment, retired or homemaker	2105	54.82
Marital status		
Currently married	3657	95.23
Single, divorced or widowed	183	4.77
	**Interquartile range (IQR)**	
Sharp score	20.00 (7.00, 56.00)	
DAS28 score	5.50 (4.00, 7.28)	
ESR (mm/h)	42.00 (22.00, 68.00)	
CRP (mg/L)	15.82 (3.61, 41.49)	
RF (U/ml)	79.15 (19.90, 195.05)	
CCP (U/ml)	132.98 (25.00, 402.67)	
IGA (g/L)	2.47 (1.87, 3.27)	
IGG (g/L)	13.45 (11.00, 16.40)	
IGM (g/L)	1.35 (0.91, 1.68)	
C3 (g/L)	112.45 (96.50, 129.30)	
C4 (g/L)	24.7 (19.20, 30.80)	
BALP (ng/ml)	660.13 (431.37, 851.60)	
BGP (ng/ml)	3479.49 (2871.84, 4825.99)	
OPG (ng/ml)	117.20 (948.12, 1338.62)	
RANKL (ng/ml)	1085.40 (955.51, 1322.46)	

Demographic and clinical variables of all participants were presented in mean (standard deviation), percentage or median (interquartile range)

### Spearman correlation analysis of sharp score and clinical indexes

**Fig. 1:**
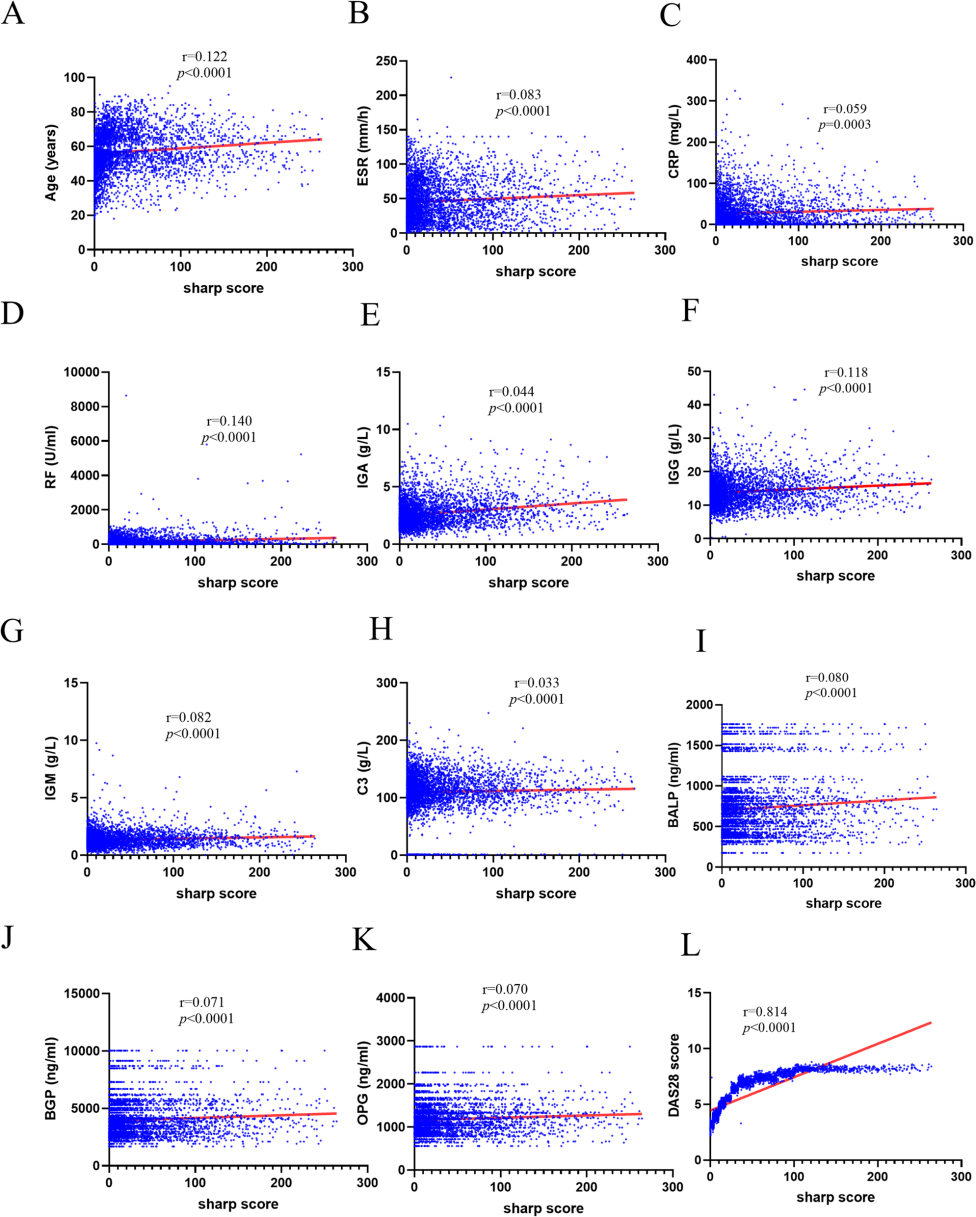
Spearman correlation analysis of sharp score and clinical indexes. Spearman correlation analyses were probed between sharp score with Age, ESR, CRP, RF, IGA, IGG, IGM, C3, BALP, BGP, OPG, and DAS28, respectively. R and *p* values were attached to each panel

To determine whether correlations existed between sharp score and clinical indexes, a Spearman correlation test was performed. Age, ESR, CRP, RF, IGA, IGG, IGM, C3, BALP, BGP, OPG, DAS28 were all positively correlated with sharp score, as seen in Fig. 1.

### Association rules analysis of sharp score and clinical indexes

Association rules analysis of sharp score and clinical indexes can be found in Table 2. Set the minimum support to 80% and the minimum confidence to 80%. Through Aprior module analysis, the correlation between sharp score and clinical indexes was obtained, and the degree of lift was more than 1 and *P*<0.05.

**Table 2:** Association rules analysis of sharp score and clinical indexes

**Items (LHS ⇒ RHS)**	**Support**	**Confidence**	**Lift**	***P* value**
{sharp score ↑} ⇒ {ESR ↑}	83.68%	91.72%	1.05	<0.01
{sharp score ↑} ⇒ {CRP ↑}	83.05%	91.03%	1.04	<0.01
{sharp score ↑} ⇒ {RF ↑}	81.25%	91.03%	1.04	<0.01
{sharp score ↑} ⇒ {IGA ↑}	83.05%	87.71%	1.05	<0.01
{sharp score ↑} ⇒ {IGG ↑}	87.67%	87.55%	1.05	<0.01
{sharp score ↑} ⇒ {C3 ↑}	81.25%	87.55%	1.05	<0.01
{sharp score ↑} ⇒ {BGP ↑}	83.68%	87.04%	1.05	<0.01
{sharp score ↑} ⇒ {RANKL ↑}	81.25%	86.79%	1.05	<0.01
{sharp score ↑} ⇒ {DAS28 ↑}	81.25%	86.34%	1.05	<0.01

Association rules analyses were performed between sharp score and multiple variables using Aprior module analysis for correlations. The minimum support and the minimum confidence were set to 80%. The degree of lift was set to > 1 and a *P* value<0.05 was considered significant

### Logistic regression analysis of sharp score and clinical indexes

**Fig. 2:**
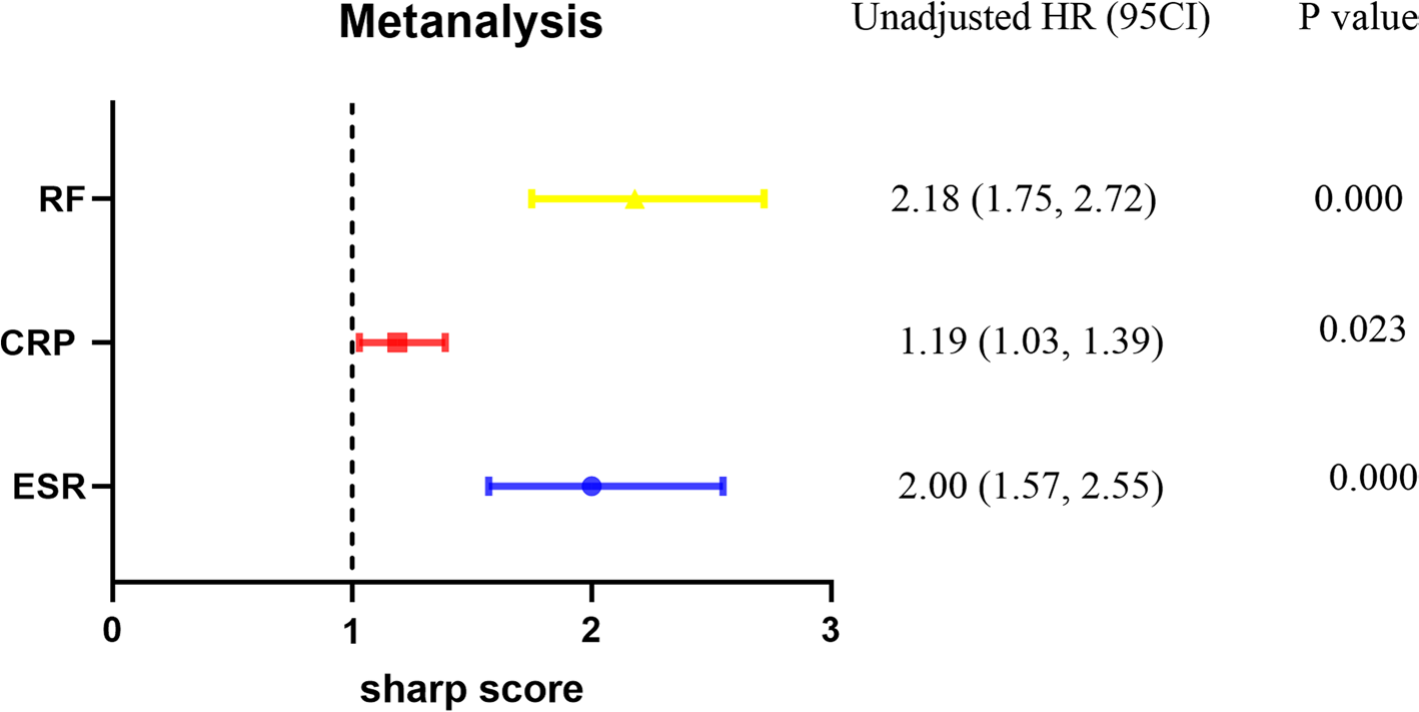
Logistic regression analysis of sharp score and clinical indexes. Logistic regression analysis results showed three risk factors for sharp score: RF, CRP, and ESR

Logistic regression analysis of risk factors of sharp score was carried out. Significant differences in sharp score were found between RA patients with ESR (*p* = 0.000), CRP (*p* = 0.023), RF (*p* = 0.000), indicating that ESR, CRP, RF were risk factors for sharp score, the higher expression of ESR, CRP, RF, the high score of sharp (Fig. 2).

### Comparison of sharp score among different variables

As shown in Table 3, there was a higher sharp score of female patients compared to male (21.00, IQR (7.00, 60.13) vs 17.00 (IQR (7.00, 42.13)) and a high sharp score of over 50 years old compared to under 50 years old (24.00, IQR (10.00, 60.13) vs 10.00 (IQR (3.00, 45.00)). There was a higher sharp score of RF-positive compared to RF-negative (23.00, IQR (8.00, 64.63) vs 13.00 (IQR (4.38, 35.63)). There was a higher sharp score of ever-smoker compared to never-smoker (20.00, IQR (7.50, 56.50) vs 19.50 (IQR (4.50, 55.00)).

**Table 3:** Comparison of sharp score among different variables

**Quantitative Variables**	**Group**	**Sharp score**	**Z**	***P* value**
Sex	Female	21.00 (7.00, 60.13)	9.482	0.002
	Male	17.00 (7.00, 42.13)		
Age	<50 years	10.00 (3.00, 45.00)	154.33	0.000
	≥50 years	24.00 (10.00, 60.13)		
Symptom duration	<5 years	5.5 (0.00, 1.50)	443.97	0.000
	≥5 years	25 (10.50, 64.00)		
DSA28 score	<3.2	0.50 (0.00, 1,50)	2813.43	0.000
	≥3.2<5.1	6.50 (3.50, 10.00)		
	≥5.1	47.00 (25.50, 89.00)		
RF	Positivity	23.00 (8.00, 64.63)	94.01	0.000
	Negativity	13.00 (4.38, 35.63)		
CCP	Positivity	20.00 (7.00, 57.38)	3.027	0.082
	Negativity	17.50 (5.50, 47.00)		
Ever smoker	Yes	20.75 (4.50, 55.50)	0.996	0.318
	No	19.50 (7.00, 56.50)		
Ever drinker	Yes	20.00 (7.50, 56.50)	3.827	0.050
	No	19.50 (4.50, 55.00)		
Radiographic grading of hands	I	1.50 (0.50, 2.50)	3546.75	0.000
	II	10 (6.50, 14.00)		
	III	28.50 (23.00, 37.00)		
	IV	87.00 (61.50, 122.50)		

Subgroup analyses for sharp score, according different variables (Sex, Age, Symptom duration, DSA28 score, RF, CCP, smoking history, drinking history, Radiographic grading of hands)

## Discussion

This study was a large-sample retrospective study, which has characterized sharp score and its effective factors in RA. The role of DAS28, clinical indicators, bone metabolism markers, and sociodemographic factors as determinants of sharp score was examined. Age, ESR, CRP, RF, IGA, IGG, IGM, C3, C4, BALP, BGP, OPG, RANKL, DAS28 were associated with sharp score. ESR, CRP, RF were also risk factors of sharp score.

Joint damage is very common in the early stage of RA, even within 2 years following disease onset in most patients (70–93%) [17, 18]. Therefore, the probability of erosions occurring early in RA is adequately high [8, 19]. Therefore, joint damage can trigger generate and maintain pain, which is a principle cause of disability and functional decline [20]. The research conducted by Corbett et al. manifested that the occurrence of hand erosions in the first 2 years of RA was the strongest predictor of the dysfunction after 15 years [21]. Early quantitative assessment of joint destruction and bone erosion are the first step to prevent or decrease its damage [22, 23].

In spite of the lacking of a similar study so far depicting sharp score and its effective factors in RA, a few studies have described sharp score as an important observation index and effective factor of RA [24]. LMAJansen followed early RA patients for 1 year, concluded that progression of these lesions was predicted by the number of radiographic lesions and Sharp/van der Heijde score [12]. Similar findings were also observed in a cross-sectional research of RA patients with secondary SS (sSS) by Lindsay E. Brown et al., which found that RA patients with sSS exhibiting worse joint damage was associated with higher sharp score [13]. As a part of our ongoing research on the joint destruction and bone erosion, in the present study we focused on sharp score, which might have significant diagnostic value for RA.

As reflected by analyses of Spearman correlation and Association rules, considerable positive correlations were noted between age, ESR, CRP, RF, IGA, IGG, IGM, C3, BALP, BGP, OPG, DAS28 and sharp score in our work. In addition, Logistic regression analysis elucidated ESR, CRP, and RF as risk factors for the sharp score. DAS28, clinical indicators, bone metabolism markers, and sociodemographic factors differences in sharp score outcomes remains enigmatic in the China and little has been known about the influence of sharp score. There are differences in sharp score of different genders, which showed that a higher sharp score of female patients compared to male [25]. There are different explanations of these gender-based differences, which may be the biological progression of disorders and self-perception and reporting of symptoms [26]. Higher DAS28, RF^+^ and radiographic grading were also associated with sharp score, which can be attributable to its link to facilitated inflammation and comorbidities [27, 28]. Symptom duration and smoking history could affect sharp score progression by changing medication adherence, health literacy, and self-care [29, 30].

Several strengths exist in our study. Initially, our work is the first retrospective research of sharp score in RA patients in China, and has a unique position in ascertaining the effective factors of sharp score in China. Secondly, as we know, this is the only research so far that has utilized three methods to identify a significant correlation between sharp score and different variables. One of the limitations of our study was the lack of multi-center and inclusion of diverse ethnic/racial groups. Furthermore, the sample size in our research is small, which may limit the discovery of remarkable differences between sharp score and different variables. Additionally, we also need to study the diagnostic accuracy and importance of magnetic resonance imaging and take it into the next research.

There is no doubt that some limitations of the current study should be realized. Firstly, although the sample size of the current study is big, it yet derived from a single-center trail, where patients were all retrospectively enrolled. It’s necessary to validate and further explore the performance of sharp score in prospective cohorts nationwide or even worldwide. The lack of a validation cohort inevitably undermines the scientific power of our study. Secondly, patients included in our study may not be representative of the general RA population in China, hence a spectrum bias may exist here. Last but not least, we do admit that the multiple r values lower than 0.2 in this study could only indicate weak correlations, despite the statistical significances we observed. However, we also believe the large-scale profile of the current study could offer insights for future researches.

In conclusion, the study has characterized the correlation of sharp score with DAS28, clinical indicator, bone metabolism markers, and sociodemographic factors, and illustrated a significant correlation between sharp score and these variables. The demonstrated differences in joint damage and bone erosion in the early stage of RA may afflict the following long-term outcomes. During the early stage of the disease, differences in sharp score should be considered in developing treatment options when patients are mostly likely to benefit from interventions. This assumes a pivotal role in improvement of disease control and functional outcomes.

## Conclusions

In summary, the sharp score in patients with RA was significantly increased and closely related to disease activity. In addition to that, ESR, CRP and RF were risk factors for sharp score. So, sharp score should be considered in developing treatment options for RA.

## Data Availability

The raw data supporting the conclusions of this article will be made available by the authors, without undue reservation to any qualified researcher. For access to study data please contact the corresponding author.

## Abbreviations

RA: Rheumatoid arthritis
DAS28: Disease Activity Score
CRP: C-reactive protein
RF: Rheumatoid factor
ESR: Erythrocyte sedimentation rate
CCP: Anti-cyclic citrullinated peptide antibody
IGA: Immunoglobulins A
IGG: Immunoglobulin G
IGM: Immunoglobulin M
C3: Complement 3
C4: Complement 4
BALP: Bone alkaline phosphatase
OC: Osteocalcin
OPG: Osteoprotegerin
RANKL: Receptor activator for nuclear factor-κB ligand
ARM: Association rule mining
